# IL-10 plays an important role in the control of inflammation but not in the bacterial burden in *S. epidermidis* CNS catheter infection

**DOI:** 10.1186/s12974-016-0741-1

**Published:** 2016-10-13

**Authors:** Yenis M. Gutierrez-Murgas, Gwenn Skar, Danielle Ramirez, Matthew Beaver, Jessica N. Snowden

**Affiliations:** 1 Department of Pediatrics, 985900 Nebraska Medical Center, University of Nebraska Medical Center, Omaha, NE 68198-5900 USA; 2 Pathology and Microbiology, University of Nebraska Medical Center, Omaha, NE 68198 USA; 3 Pediatric Residency Program, Baylor College of Medicine, San Antonio, TX 78207 USA

**Keywords:** *S. epidermidis*, IL-10, Central nervous system, Cytokines, Chemokines, Biofilm, Catheter, Shunt

## Abstract

**Background:**

Shunt infection is a frequent and serious complication in the surgical treatment in hydrocephalus. Previous studies have shown an attenuated immune response to these biofilm-mediated infections. We proposed that IL-10 reduces the inflammatory response to *Staphylococcus epidermidis* (*S. epidermidis*) CNS catheter infection.

**Methods:**

In this study, a murine model of catheter-associated *S. epidermidis* biofilm infection in the CNS was generated based on a well-established similar model for *S. aureus*. The catheters were pre-coated with a clinically derived biofilm-forming strain of *S. epidermidis* (strain 1457) which were then stereotactically implanted into the lateral left ventricle of 8-week-old C57BL/6 and IL-10 knockout (IL-10 knockout) mice. Bacterial titers as well as cytokine and chemokine levels were measured at days 3, 5, 7, and 10 in mice implanted with sterile and *S. epidermidis-*coated catheters.

**Results:**

Cultures demonstrated a catheter-associated and parenchymal infection that persisted through 10 days following infection. Cytokine analysis of the tissue surrounding the catheters revealed greater levels of IL-10, an anti-inflammatory cytokine, in the infected group compared to the sterile. In IL-10 KO mice, we noted no change in bacterial burdens, showing that IL-10 is not needed to control the infection in a CNS catheter infection model. However, IL-10 KO mice had increased levels of pro-inflammatory mediators in the tissues immediately adjacent to the infected catheter, as well as an increase in weight loss.

**Conclusions:**

Together our results indicate that IL-10 plays a key role in regulating the inflammatory response to CNS catheter infection but not in control of bacterial burdens. Therefore, IL-10 may be a useful therapeutic target for immune modulation in CNS catheter infection but this should be used in conjunction with antibiotic therapy for bacterial eradication.

## Background

Cerebrospinal fluid shunt placement for the treatment of hydrocephalus is one of the most common procedures performed by pediatric neurosurgeons in the USA, with tens of thousands of shunts implanted annually [1]. Unfortunately, 30–40 % of all shunts placed in pediatric patients fail within the first year, resulting in a shunt revision to a primary placement ratio of 3:1 in many health-care centers [1, 2]. One of the most common causes of shunt failure is infection, reported in 5–30 % of cases [2]. This equates to approximately 2400 hospital admissions each year, with an increase in seizure frequency and intelligence quotient (IQ) loss in many children, who represent the highest risk population for these infections [2–4]. The most common organism responsible for shunt infections, *Staphylococcus epidermidis* (*S. epidermidis*), is known to form biofilms, which are communities of bacterial cells that aggregate on the catheter surface, encased in a protective self-produced matrix [2, 5]. The biofilm’s recalcitrance to antimicrobial agents makes it difficult to manage central nervous system (CNS) catheter infections non-surgically, such that catheter removal is currently required for effective treatment [2, 6]. Studies designed to advance our understanding of the unique immune response to shunt infections in children are needed to develop improved diagnostic, treatment, and prevention strategies for these serious infections.

Our laboratory developed a murine model of CNS catheter infection to generate a consistent catheter-associated infection with *Staphylococcus aureus* (*S. aureus*), mimicking what is seen in humans with ventricular shunt infections [7]. Using this model, we have been able to demonstrate a relative decrease in inflammation in biofilm infections in the brain, as compared to abscess or infection with biofilm-deficient strains of bacteria [8]. This suggests that there is an alteration in the immune response to biofilm infections, possibly contributing to the persistence of these infections in patients. One potential cause of the skewed immune response to these biofilm infections is an increase in interleukin-10 (IL-10), which may play a role in regulating inflammatory responses in this setting. IL-10 is a potent anti-inflammatory cytokine that can be produced by both innate and adaptive immune cells [9]. IL-10 polymorphisms in humans and mice have been associated with an increase in autoimmune disease, such as inflammatory bowel disease, atopic dermatitis, and wheezing, as well as an increased inflammatory response to some microbial pathogens [9–12]. Interestingly, this increase in inflammatory response to microbes does not necessarily result in increased clearance of the organism, suggesting that the role of IL-10 in response to infection is likely pathogen-specific [9]. Very little has been studied about the role of IL-10 in response to *S. epidermidis* infection or biofilm infections specifically, but elevated levels of IL-10 have been associated with poor outcomes in patients with *S. aureus* bacteremia, suggesting this cytokine may play a role in staphylococcal disease [12]. In these studies, we have adapted our mouse model of *S. aureus* CNS catheter infection to generate infection with *S. epidermidis* and have used this model to define the contribution of IL-10 to the inflammatory response to CNS catheter infection. Our results show that alterations in IL-10 result in increases in inflammatory mediators and infiltration of peripheral immune cells but do not significantly impact bacterial burdens.

## Methods

### Mouse strain

All in vivo experiments were performed using 8- to 9-week-old male C57BL/6 or IL-10 knockout (IL-10 KO) mice (The Jackson Laboratory, Bar Harbor, ME). Each experiment was done independently at three different dates with four to five mice per experimental group (*n* = 12–15 mice/group). The protocol for animal use was approved by the University of Nebraska Medical Center Institutional Animal Care and Use Committee (protocol #09-053-08-FC) and is compliant with the National Institute of Health (NIH) guidelines for the use of rodents.

### Bacterial strain

*S. epidermidis* 1457 was graciously provided by Dr. Paul Fey laboratory (University of Nebraska Medical Center). This isolate was recovered from an infected central venous catheter and initially characterized by Mack et al. [13] and has not been laboratory adapted or modified since its initial characterization.

### Catheter preparation and implantation

Hollow bore silicone catheters (2 mm in length, 1 mm in diameter; Cook Medical Inc., Bloomington, IN) were incubated with *S. epidermidis* 1457 overnight to ensure bacterial adherence to the catheter, resulting in reproducible catheter colonization of approximately 6 × 10^7^ cfu/catheter. *S. epidermidis* required a 4-log higher inoculum in order to establish infection, in comparison with our prior work in *S. aureus*, reflecting the difference in virulence of these strains [7]. Specifically, the primary virulence property associated with *S. epidermidis* is its ability to form biofilm, as it lacks the production of virulence factors seen in *S. aureus* [14]*.* While higher than the bacterial inoculum used in our prior *S. aureus* studies, this is the lowest bacterial inoculum tested that generated consistent infection, in order to most closely replicate natural disease in which small exposures of bacteria at the time of surgery or subsequent access to the catheter are believed to cause infection. Subsequently, catheters were stereotactically implanted in the left lateral ventricle of anesthetized mice as previously described [7, 8]. In brief, mice were anesthetized with intraperitoneal (i.p.) injection of ketamine and xylazine (100 to 200 mg/kg and 5 to 13 mg/kg, respectively) and a 1-cm longitudinal incision was made in the scalp to expose the skull sutures. The catheter was placed vertically into the left ventricle as previously described via a burr hole in the skull placed using a rodent stereotactic apparatus (Stoelting Co., Wood Dale, IL). To secure the catheter in place and reduce bacterial efflux and bleeding, bone wax was used to seal the burr hole and Vetbond surgical glue to close the incision (3M, St. Paul, MN). Animals tolerated the procedure well with <1 % mortality. Animals were weighed daily following surgery to assess weight change as a symptom of illness.

### Bacterial enumeration from catheters and associated brain parenchyma

Catheters and associated brain tissue were collected at the designated time points post-surgery in four to five mice per group at each time point. Briefly, catheters were rinsed in sterile phosphate-buffered saline (PBS) after removal to eliminate non-adherent bacteria and the biofilms sonicated for 5 min in 500 μl PBS. Tissue within 1 mm of initial catheter placement was homogenized in 500 μl sterile PBS supplemented with a complete protease inhibitor cocktail tablet (Roche, Basel, Switzerland) and RNase inhibitor (Promega, Madison, WI) using a Polytron homogenizer (Brinkmann Instruments, Westbury, NY). This tissue is referred to as “immediately adjacent tissue” hereafter. A 100-μl aliquot of these homogenates and supernatants from sonicated catheters were used to quantitate bacterial titers via tenfold serial dilutions in tryptic soy agar plates. These homogenates were also used to determine cytokine/chemokine levels after centrifugation and storage at −80 °C as previously described [7, 8].

### Cytokine/chemokine analysis

Protein concentrations from brain samples were measured using a bicinchoninic assay (BCA; Thermo Fisher Scientific Inc., Rockford, IL) and used to normalize cytokine levels to correct for any variation in tissue sample size. Inflammatory mediators were measured with mouse microbead array system according to the manufacturer’s instruction (MILLIPLEX; EMD Millipore Corp., Billerica, MA). This assay allowed for simultaneous measurement of 12 different inflammatory molecules in a single 50-μl supernatant sample including (IL-1β, IL-4, IL-6, IL-9, IL-10, IL-12p70, IL-13, CXCL1, CXCL2, CXCL9, CXCL10, and CCL2). Results were analyzed using Multiplex Assay Analysis Software and normalized to tissue protein level as measured by BCA.

### Statistical analysis

Analysis of significant differences between experimental groups was determined using unpaired Student’s *t* test with SigmaStat (SPSS Science, Chicago, IL) at the 95 % confidence interval. A *p* value of less than 0.05 was considered statistically significant.

## Results

### Catheter-associated bacterial growth predominates during the initial post-implantation period, consistent with biofilm formation

**Fig. 1:**
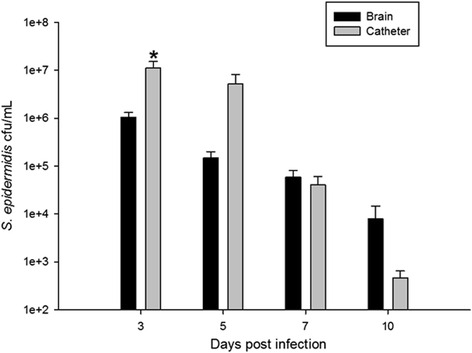
*S. epidermidis* generates a catheter-associated infection in the CNS. This pattern of growth is consistent with biofilm infection. The cultures from the catheters and parenchyma of the mice implanted with sterile catheters were negative and are not presented in this graph. *p < 0.05

To assess the bacteriologic and inflammatory kinetics of *S. epidermidis* catheter-associated infection in the CNS, mice were implanted with silicone catheters pre-coated with *S. epidermidis* 1457. At designated time points following implantation, the catheters were removed and adherent biofilm burdens enumerated for comparison with the bacteria isolated from the immediately adjacent tissue. As observed in Fig. 1, higher bacterial burdens were measured after sonication of infected catheters at days 3 and 5 post-surgery, contrasted with the lower bacterial growth observed in the immediately adjacent tissue. This is consistent with biofilm infection and with prior experiments utilizing *S. aureus*, in which the higher colony counts in association with the catheter were confirmed to be visibly consistent with biofilm formation on electron microscopy [7]. This model was capable of reproducing *S. epidermidis* catheter-related infection demonstrating its appropriateness to adequately evaluate host immune response to *S. epidermidis* infection in the CNS.

### *S. epidermidis* central nervous system catheter infection induces skewed production of pro- and anti-inflammatory mediators in the brain

**Fig. 2:**
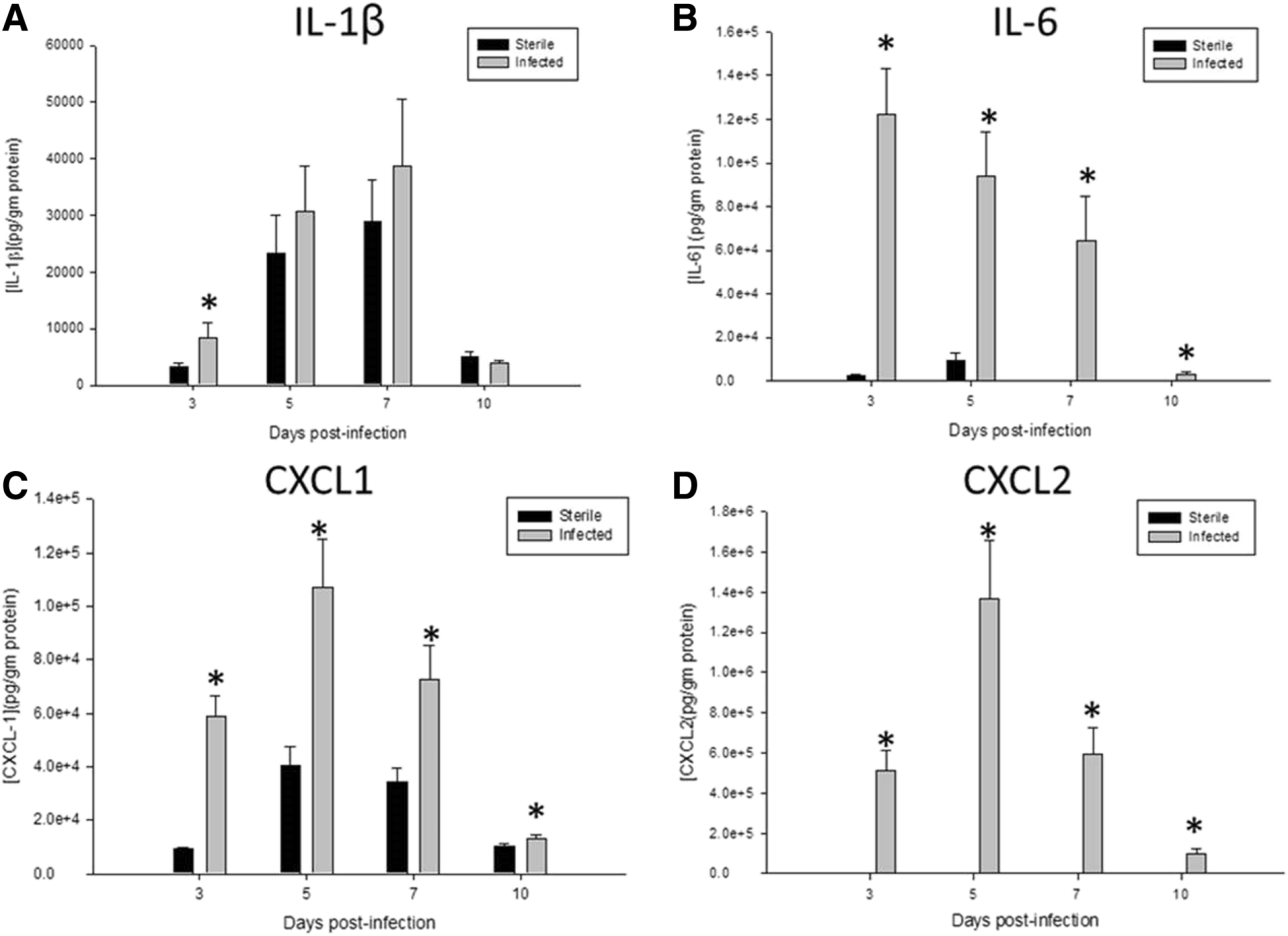
An increase in certain pro-inflammatory chemokines and cytokines is observed, similar to previous studies with *S. aureus*. Catheter-associated infection in the CNS with *S. epidermidis* results in an increase in pro-inflammatory cytokines (IL-1β (**a**); IL-6 (**b**) and chemokines (CXCL1 (**c**); CXCL2 (**d**). Results were normalized to the amount of total protein recovered to account for differences in tissue sampling size. *p < 0.05

To assess the inflammatory response to *S. epidermidis* catheter infection in the CNS, homogenates of immediately adjacent catheter-associated tissue were analyzed for IL-1β, IL-6, CXCL2, and CXCL1 production by multianalyte microbead array (Fig. 2). IL-1β was slightly, but significantly, elevated at day 3, and IL-6 was significantly increased at multiple time points than the infected group compared to the uninfected mice. CXCL1 and CXCL2, the chemokines responsible for early phase cellular recruitment, were also increased in the tissues surrounding the infected catheters, suggesting that innate immune cells may be recruited as part of the inflammatory response to *S. epidermidis* infection in the brain. The pro-inflammatory environment observed in this model is congruent with results obtained with similar studies using *S. aureus* ACH1719 strain [7, 8]. No differences were noted in levels of Il-4, IL-9, IL-13, CXCL9, or CCL2 (data not shown).

**Fig. 3:**
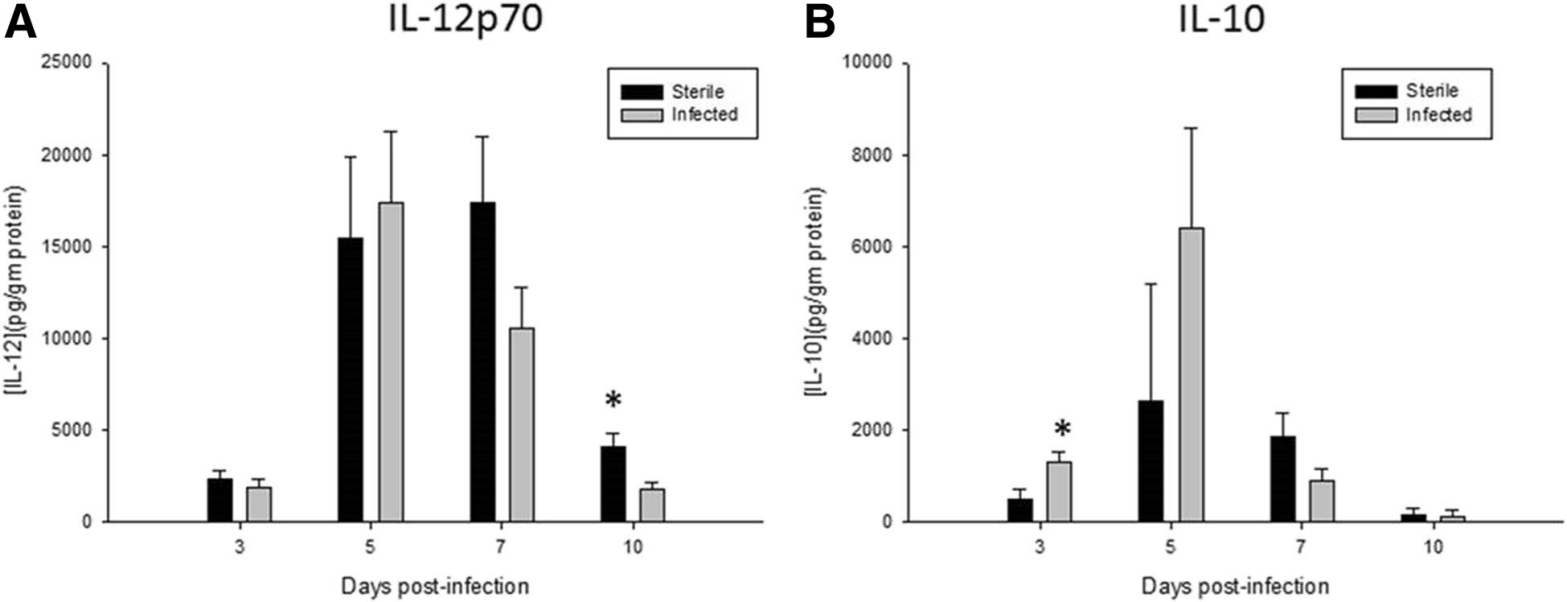
There is attenuation of the inflammatory response to *S. epidermidis* in a catheter infection in the brain, with no increase in IL-12p70 (**a**) and an increase in anti-inflammatory cytokine IL-10 (**b**). This demonstrates a skewed immune response to *S. epidermidis* distinct from that previously reported with *S. aureus.* Results were normalized to the amount of total protein recovered to account for differences in tissue sampling size. *p < 0.05

In contrast to in vitro and in vivo studies utilizing *S. aureus*, the pro-inflammatory cytokine IL-12p70 was not elevated following the infection with *S. epidermidis* in this model [15–18]. There was no significant difference between levels of IL-12p70 in the immediately adjacent tissues surrounding the infected versus uninfected catheters (Fig. 3a). The anti-inflammatory cytokine IL-10 was slightly elevated in response to *S. epidermidis* infected catheters, demonstrating a potential role for anti-inflammatory as well as pro-inflammatory pathways in response to CNS catheter infection (Fig. 3b).

### IL-10 is not required to support *S. epidermidis* infectivity but does play a significant role in regulating the immune response to infection

**Fig. 4:**
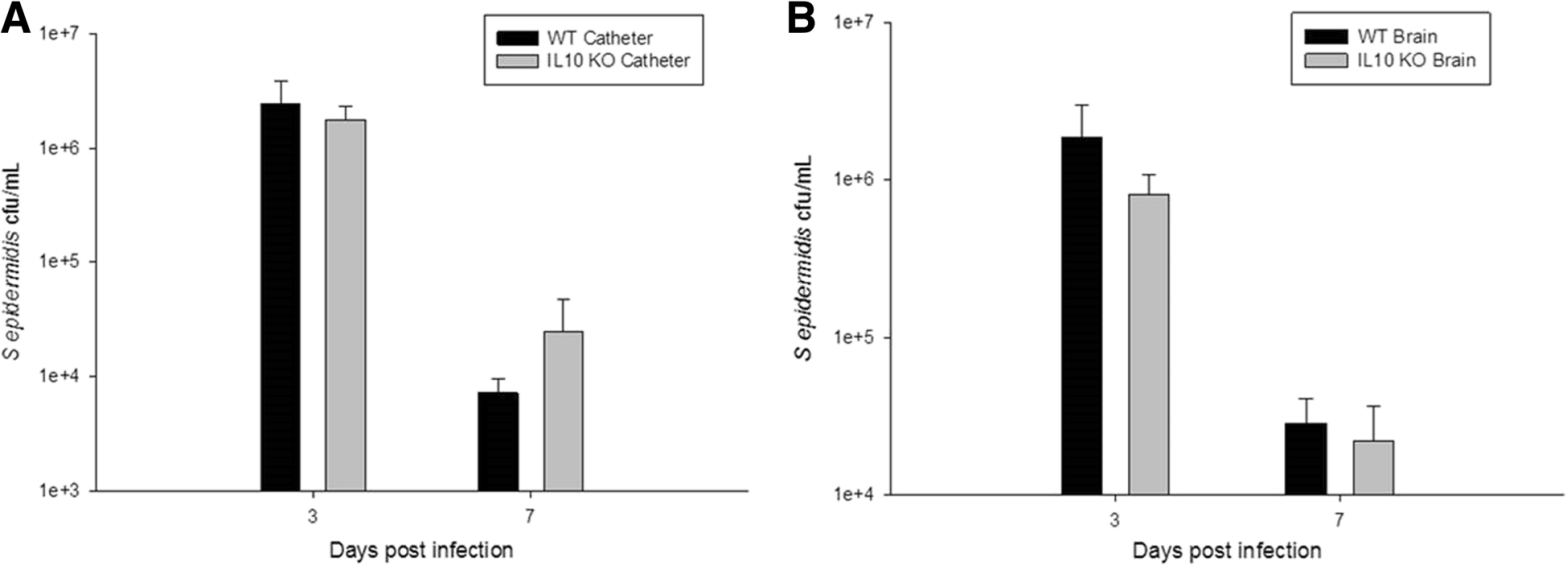
IL-10 is not needed for *S. epidermidis* to establish catheter-associated infection in the central nervous system. There was no significant difference between catheter-associated (**a**) or immediately adjacent tissue (**b**) bacterial burdens in the wild-type (*black bars*) versus IL-10 KO (*gray bars*) mice at days 3 and 7 following infection. Interestingly, 2 of the 12 IL-10 KO mice had no detectable bacteria in the immediately adjacent tissue or along the catheter at day 7, while all 12 wild-type mice had measurable bacteria at that time

**Fig. 5:**
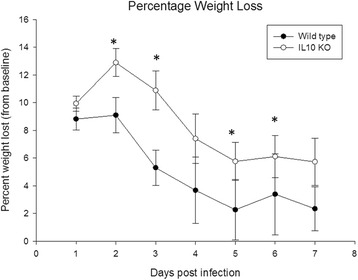
IL-10 KO mice displayed higher weight loss than their wild-type counterparts. Mice were weighed daily, with weight loss percentage calculated per mouse per day based on starting weight. *p < 0.05

**Fig. 6:**
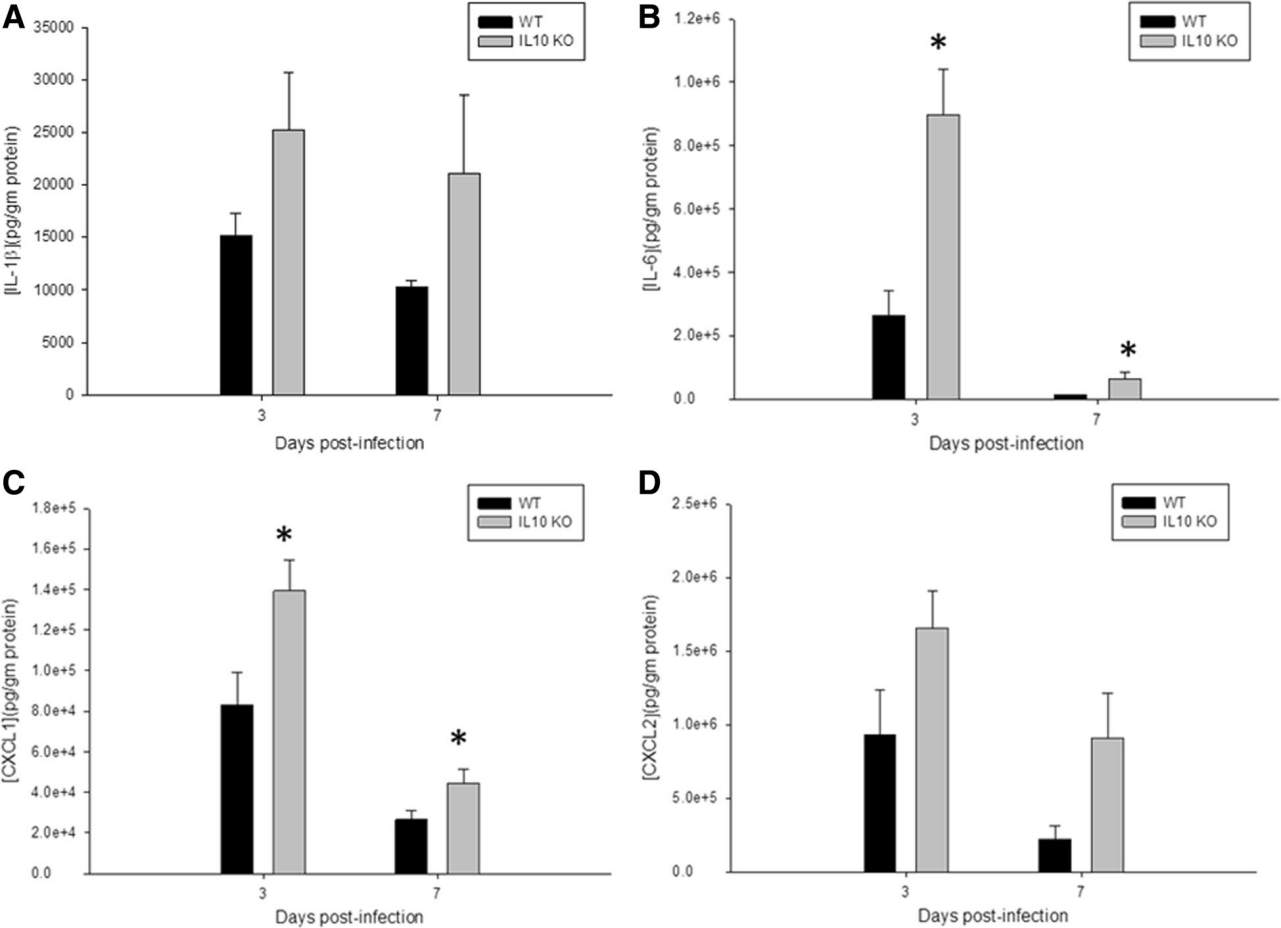
IL-10 KO mice had significantly greater production of pro-inflammatory mediators following infection despite similar bacterial burdens to wild-type mice (**a**–**d**). This may explain the greater weight loss observed in these mice and suggests that IL-10 is playing a significant role in regulating the immune response to CNS catheter infection. Results were normalized to the amount of total protein recovered to account for the differences in tissue sampling size. *p < 0.05

Given the slight increase in IL-10 noted on multianalyte microbead array (Fig. 3b), we hypothesized that the anti-inflammatory properties of IL-10 may create a permissive environment that facilitates *S. epidermidis* infection in the brain. To test this hypothesis, we implanted *S. epidermidis-*infected catheters into the CNS of wild-type C57BL/6 and IL-10 KO mice and compared their clinical response to infection, bacterial burdens, and inflammatory responses via multianalyte microbead array. Surprisingly, bacterial enumeration at day 3 and day 7 from the catheter and immediately adjacent tissue showed similar colony counts in IL-10 KO mice compared to wild-type, proving that *S. epidermidis* infectivity does not depend on the individual action of anti-inflammatory cytokine IL-10 (Fig. 4). Despite the similar bacterial burdens, however, the IL-10 KO mice showed greater evidence of clinical illness as measured by percent weight loss (Fig. 5). This difference becomes apparent by day 3 following infection, which coincides with high bacterial burdens (Fig. 4). Interestingly, this time point also coincides with pro-inflammatory mediator production at day 3, which was significantly increased in the IL-10 KO mice (Fig. 6). The IL-10 KO mice had significantly higher levels of IL-6 and CXCL1 in the immediately adjacent tissue surrounding the infected catheters, with a trend toward higher levels of IL-1β and CXCL2 as well.

## Discussion

*S. epidermidis* and *S. aureus* are the most common biofilm-forming bacteria responsible for CSF shunt infections [19]. These infections hinder the effectiveness of shunt therapy and inflict an unnecessary burden in the treatment of pediatric patients, who are known to be at higher risk for shunt infection, although the mechanisms responsible for this increased risk in children are not yet defined [19]. Biofilm infections are known to be difficult to diagnose and recalcitrant to antibiotic treatment, leading to removal of the shunt [19]. This increases the numbers of surgical procedures needed for patients, in addition to increasing future infection risks as revision itself is associated with an increased risk of shunt infection [19, 20]. Infants are known to have the most adverse outcomes, such as increased risk of seizures, IQ loss, and up to 10 % death rate in patients with neurological handicap [21, 22]. Shunt infections also lead to disproportionate use of hospital days and health-care dollars [23]. Collectively, these findings highlight the significance of CNS shunt infection and the need to better understand the pathophysiology of the disease with the goal of informing better treatment strategies that would avoid the need for shunt replacement surgery.

In these studies, we adapted our established model of *S. aureus* CNS catheter infection to generate infection with the more prevalent, but less virulent *S. epidermidis* [14, 24]. While *S. epidermidis* is the cause of most CNS catheter infections, its primary virulence property is its ability to form biofilm, as it lacks the production of virulence factors seen in *S. aureus* [14]. Catheter-adherent biofilm loads registered peak values at day 3 post-surgery compared to immediately adjacent tissue in both models, consistent with biofilm formation and planktonic spread of the infection. As we have only evaluated the immediately adjacent tissue in these studies, we may underestimate bacterial spread throughout the brain. In the *S. aureus* model of infection, this pattern of bacterial growth was visually consistent with biofilm formation on electron microscopy [7]. Interestingly, both infection models demonstrate a shift to greater parenchymal involvement as overall bacterial burdens lessen in later days of infection [7]. Differences between the pathogenic natures of these two closely related species were noted by the earlier decrease in bacterial burdens and reduced mortality rate in *S. epidermidis*-infected mice compared to the prolonged course of infection and greater casualties in the *S. aureus* model [7]. Additionally, higher colony counts were necessary to achieve effective infecting doses with *S. epidermidis* (6 × 10^7^ cfu/ml) compared to *S. aureus* (8.7 × 10^3^ cfu/ml) [7]. These findings are consistent with the increased morbidity attributable to *S. aureus* in clinical settings and reflect the multiplicity of virulence factors known to be present in *S. aureus* compared to *S. epidermidis* [25]*.*

These studies also demonstrate a distinct inflammatory profile with greater evidence of attenuation in response to *S. epidermidis* biofilm infection in the CNS than was seen in our prior studies with *S. aureus* [7]*.* As in our prior studies with *S. aureus*, we observed a brief increase in inflammatory mediators at early time points following the implantation of sterile catheters which likely results from tissue damage; importantly, this inflammation is consistently less than observed in response to infected catheters [7, 8]. There is an increase in pro-inflammatory IL-1β, IL-6, CXCL1, and CXCL2 with *S. epidermidis*, demonstrating that implant-mediated *S. epidermidis* infection elicits a significant inflammatory response in the brain, similar to prior work with *S. aureus* [7]. However, we also observed a small increase in the anti-inflammatory IL-10 and a lack of pro-inflammatory IL-12p70, suggesting that there are also anti-inflammatory pathways involved in the response to *S. epidermidis* CNS catheter infection. This is similar to studies of peripheral catheter-associated infections which have demonstrated anti-inflammatory immune responses to biofilm infections, suggesting a role for biofilm structure to modulate host response [26]. Our prior studies in the brain also suggest an attenuated response to biofilm infection, with decreased levels of pro-inflammatory mediators observed when comparing biofilm infection to both parenchymal abscess infections and catheter-associated infection with a biofilm-deficient *sarA* mutant [8]. Future studies may utilize biofilm-deficient *S. epidermidis* mutants, which would allow us to better dissect if there is a distinctive effect of biofilm formation in the immune response to *S. epidermidis* as well. However, this approach may not be feasible as biofilm-deficient *S. epidermidis* may not be able to establish device-associated infection.

Herein, we report increased IL-10 levels in infected mice following *S. epidermidis* CNS catheter infection compared to the non-infected controls. This suggests a possible effect of IL-10 in curbing the CNS inflammatory environment to allow non-pathogenic *S. epidermidis* to cause infection [27, 28]. IL-10 plays a central role in chronic and acute inflammation, particularly in the CNS where prolonged contact with pro-inflammatory cytokines leads to harmful effects on neuronal function, behavior, and cognition [27, 29–32]. To further study the role of IL-10 in this model, IL-10 KO mice were infected to evaluate the bacterial kinetics and immune response in the absence of this important immune regulator. Surprisingly, IL-10 deficiency had no impact in bacterial accumulation in the catheter or immediately adjacent tissue in the first 7 days post-implantation. IL-10 may play a role in bacterial clearance in later dates post-infection, which represents a limitation of the current studies. As expected IL-1β, IL-6, CXCL1, and CXCL2 levels were increased in IL-10 KO mice. This increase in pro-inflammatory mediators likely led to the increase in weight loss in the IL-10 KO mice, as IL-1β and IL-6 have been shown to play a role in sickness behaviors [33, 34]. TNF-α also plays an important role in sickness behaviors but was not evaluated in these studies [35, 36]. Heightened levels of inflammatory cytokines and chemokines in IL-10 KO mice demonstrate that IL-10 is important in controlling immune response in this model. However, this data also shows the limitations of enhancing pro-inflammatory responses in an effort to treat CNS catheter infections, as the increased inflammation resulted not in improved bacterial clearance but rather in increased clinical illness.

While our studies have demonstrated that IL-10 is not solely responsible for dampening the immune response to permit infection to occur, it does play a role in modulating the immune response to *S. epidermidis* infection in the CNS. This may play a particularly significant role in infants, who are known to be at higher risk of CNS catheter infections [2, 4, 19]. In humans, IL-10 appears to play an important protective role in infants, with altered IL-10 levels associated with inflammatory conditions such as bronchopulmonary dysplasia, sepsis, seizures, and colitis [37–40]. Additionally, cord blood studies suggest that the neonatal immune system is primed toward increased IL-10 production, with increased IL-10 release from mononuclear cells following exposure to neonatal plasma and increased IL-10 expression from infant monocytes following TLR stimulation [41, 42]. These in vitro studies suggest that IL-10 may play an important role in regulating the immune response to infection in young hosts, which is currently being evaluated in vivo in our laboratory. Importantly, while this disease is more common in children, it is not exclusive to children and there may be distinct differences between the adult and infant responses to CNS infection such that investigation of both adult and infant models is needed.

Defining the contribution of IL-10 to pathology in vivo is essential as this will allow us to account for both the multiple antigens and evasion capabilities of clinically relevant bacteria such as *S. epidermidis*, as opposed to single antigens, in addition to the impact of immune responses on multiple cell types within the brain. The unique milieu of the CNS includes multiple mechanisms of cross talk between glia and peripheral immune cells as a means of protecting the CNS from inflammatory damage as well as invading pathogens and evaluation in vivo allows for the best opportunity to model these complex interactions. It is reasonable to suspect that IL-10 will play a greater role in immune regulation in infant hosts, given its significant role in neonatal immunity, and future studies in our laboratory will expand the current studies to this population.

## Conclusions

The difficulty in diagnosing and treating biofilm infections poses a great challenge in the control of indwelling device-associated infection in the treatment of hydrocephalus. A greater understanding of the immune mechanisms involved in these events could lead to the development of better biomarkers of infection and of increased susceptibility to infection. Additionally, immune treatment adjuvants may be developed that could aid in the treatment of these infections. Our data suggest that IL-10 may be an important target for immunomodulation in CNS catheter infection, as it plays a significant role in shaping the inflammatory response to *S. epidermidis* infection in the brain. Further in vivo studies to decipher the complicated interplay between the host and the bacterial biofilm is crucial to improve the outcomes of patients with shunt infections.

## Abbreviations

BCA: Bicinchoninic assay
CNS: Central nervous system
FACS: Fluorescent-activated cell sorting
i.p.: Intraperitoneal
I.Q.: Intelligence quotient
IL-10: Interleukin-10
IL-10 KO: IL-10 knockout
PBS: Phosphate-buffered saline
*S. aureus*: *Staphylococcus aureus*
*S. epidermidis*: *Staphylococcus epidermidis*
WT: Wild-type
